# 

**Published:** 1863-08

**Authors:** 


### INDEX.

Abstract of the Proceedings of the Buffalo Medical Association, 1, 46, 87, 124, 175, 221, 251, 298, 230, 401, 405.
Annals of the County Society of Albany,.. 282
Alcoholic Stimulants in Tuberculosis, (Dr. Flint's paper,) ........................ 14
Address—By J. A. Peters, M. D........... 289
American Medical Association,........... 490
Adulterations of Milk,.... ............ 26
Artificial Dilatation of Os and Cervix Uteri 114
Artificial Pupil—By J. F. Miner, M. D..... 1
Apoplexy, blood-letting in................ 97
Anæsthetics in Obstetrics, ................ 135
Attendance at Medical Schools,........... 190
Amputation at Shoulder Joint—By S. Barrett, M. D. ........................ 260
Acute Rheumatism, treatment of.......... 272
Annals of the Medical Society of Albany County, ............................. 280
Admission of Air into Veins—By J. F. Miner, M. D......................... 336
American Medical Association, meeting of 355
American Medical Association, notice of meeting, .............................. 400
Artificial Legs,.......................... 358
Amputation-Case in Stanton U. S. A. Hospital, ................................ 328
Abbey, O. L., M. D., web-fingered child,.. 383
Anæsthesia prolonged by injection of morphine,.............................. 389
Anæsthetics, prize essay,.................. 402
Abstract of the Transactions American Medical Association,.............436 467
A Suggestion,............................ 461

Belladonna in Epilepsy.................. 28
Bowman's Medical Chemistry,............ 38
Bromine in Hospital Gangrene—By W. B. Alley, M. D............................ 41
Blood-letting in Apoplexy,................ 97
Brownell, Charles E., M. D., death of..... 159
Buffalo Dispensary appointments,........ 196
Butler, Wm. H., M. D., report of cases in Stanton Military Hospital, .......179, 247
Butler, Wm. H., cases of aphonia, ....... 214
Butler, Wm. H., hospital cases,........... 325
Butler, Wm. H., M. D., death of.......... 277
Berkshire College Commencement,........ 235
Bite of Rattlesnake—By Wm. Foster,..... 383
Bartlett, Dr. against Erie County Medical Society,......................... 393, 416
Burns, ................................. 443
Barrett, S., M. D., amputatiou shoulder joint,.............................. 260
Bumstead on Venereal Disease........... 474

Communication from J. F. Norton,..... 9
Cholera, sub-cutaneous injections in...... 19
Calomel and Tartar Emetic as remedial agents—exclusion from supply table,.. 32
Commencement of Lectures in the Berkshire Medical College. ............... 40
Cases in Stanton Military Hospital—By W. H. Gail,.............................. 49, 85
Cholera Infantum,........................ 52
Cataract, operations for .................. 62
Canada Medical Journal.................. 479
Cataract by extraction—By J. F. Miner, M.D..................................... 3
Courts and Medical Societies,............63, 91
Commission on the colored population,.... 110
Carson, Joseph M. D., Materia Medica and Pharmacy,........................... 157
Clinical Remarks upon Surgical Cases—By J. F. Miner, M. D......176, 217, 261, 307
Cases in Union Chapel Hospital—By Wm. H. Butler, M. D.................. 179, 247
Cases of Aphonia—By Wm. H. Butler, M.D. 214
Camp Diseases—By J. J. Woodard, M. D... 278
Chromolithographic Eye-ground,......... 280
Consumption contagious—By W. M. Cornell, M. D., L. L. D. ................. 461
Commencement in the University of Buffalo, .................................. 310
Correction.............................. 324
Cases in Stanton U. S. A. Hospital,....... 328
Castor Oil rendered tasteless,............. 440
Confederate Medical Journal,............. 392
Cystic Tumor of the Neck—By J. F. Miner, M. D..... ....................... 179
Cystic Degeneration of Mammary Gland—By J. F. Miner, M. D.... ........... 377
College Commencements................. 364
Completion third volume................. 471

Delirium Tremens...............27, 256, 265
Dilatation Os and Cervix Uteri............ 114
Drugs in the Treatment of Disease......... 147
Draft in Erie County...................... 159
Directions for restoring the Drowned...... 188
Dysentery in Ohio—By Andrew J. Scott, M. D.................................. 241
Diphtheria .......... ...................47, 270
Dalton's Treatise on Physiology........... 360
Diabetes—By James P. White, M. D....... 373
Diabetes, diet in.......................... 388
Deaths, report of..................40, 402, 444

Examination of Urine.................. 20
Epilepsy, belladonna in........... ........ 28
Epilepsy, report upon..................... 162
Eastman, Sandford, M. D., presentation to. 194
Excision of globe of Eye—By J. F Miner, M. D.................................. 217
Exsection head of humerus,............... 307
Erie County Medical Society.............. 240
Erie County Medical Society, semi-annual meeting .............................. 440

Epilepsy—By William M. Cornell, M. D.... 350
Ellis' Medical Formulary—By R. P. Thomas M. D.... 361
Eleventh Annual Meeting Illinois Medical Society.... 361
Etherization, death from.... 364
Eastman, Sandford, report of deaths, 40, 402, 444, 480.

Fistula Vesico Vaginal.... 28
Fistula in Ano—By J. F. Miner, M. D..... 330
Fistula in Ano—By T. F. Rochester, M. D. 332
Field and Hospital Stretcher.... 118
Foreign body removed from the left bronchus—By J. F. Miner, M. D.... 129
Fallacy of the Change Type Theory.... 343
Fibrinous Coagula in the Heart.... 355
Foreign bodies in the eye.... 127
Fracture and removal of the head of thigh bone—By J. F. Miner, M. D.... 299

Gonorrhœa.... 29
Gastrotomy—By John O'Reilly, M. D., F. R. C. S. I.... 76
Gun-shot wounds of the Badder—By W. H. Butler, M. D.... 456
Gun-shot wound, fracture of joint—By E. N. B. Smith,.... 460
Gun-Shot Wounds—By Wm. H. Gail.... 121
Goldsmith, M., Gangrene Erysipelas and Pyemia.... 154
Gould, William, M. D., Intussusception.... 303
Gail, Wm. H., M. D., Gun Shot Wounds, 121, 328
Gordon's Vaccinator.... 369
Glanders—By Robert Jennings.... 362
Gay, C. C. F., M. D., Fractures,.... 251

Hernia, Taxis in.... 30
Hernia Femoral.... 31
Hamilton on Fractures and Dislocations.... 38
Hospital Gangrene—By W. B. Alley, M. D.. 41
Hammond's Military Hygiene.... 69
Haywood, Dr. George, Death of.... 120
Hospital Gangrene, Erysipelas and Pyaemia—By M. Goldsmith.... 154
History of the War for the Union.... 158
Hygiene, Mental, J. Ray, M. D.... 198
Hand-Book of Practice—By Wm. Elmer.... 236
Hæmatocele Retro-Uterina—By H. Lassing, M. D.... 282
Health Physician's Report and Report of Deaths.... 40, 321, 402, 444
Hospital Cases, from the Note-Book of the late W. H. Butler, M. D.... 325
Heart Clot—By J. F. Miner, M. D.... 10
Hodge on Obstetrics,.... 477
Miner, M. D.... 10

Intracardiac Blood Concretions—By J. F.
Indolent and Sloughing Ulcers—By J. F. Norton, M. D.... 9
Iodide of Lime.... 18
Insanity, Hydrocyanic Acid in.... 29
Injections in Gonorrhœa.... 29
Insane Asylum.... 66
Internal Revenue Tax by Physicians.... 80
Iridectomy—By Julius Homberger, M. D.. 231
Intussusception of the Colon—By Wm. Gould, M. D.... 303
Iridectomy, James Syme on.... 348
Iridectomy, B. Joy Jeffries, Boston.... 349
Iridectomy—By J. F. Miner, M. D.... 2

Jenks, E. W., M. D., Spotted Fever.... 81
Jones, T. Wharton, F. R. S., Opthalmic Medicine and Surgery.... 196

Krombein, M. D., Cases of Trichinous Disease.... 430

Lime—Iodide of.... 18
Laceration of Perineum.... 30
Literary Journals.... 117, 118
Lind University.... 129
Lectures in University of Buffalo.... 152
Lassing, H., M. D., Translation of Report upon Epilepsy.... 161, 201
Lassing, H., M. D., Translation from the German—Retro-Uterine Hæmatocele... 282
Lithotomy.... 349
Lothrop, J. R., M. D., Trichina Spiralis... 430
Lockwood, T. T., M. D., Cystic degeneration of testicles,.... 221

Morphia—Subcutaneous Injection.... 19
Milk—Adulterations.... 26
Medical Society, Erie County.... 35
Medical Student's Vade Mecum.... 36
Medical Societies and Courts.... 63
Maternal Impressions.... 103
Meningitis.... 105
Medical Department of Army.... 106
Manual of Instruction for Enlisting Soldiers—By Robert Bartholow, M. D.... 111
Modus Operandi of Medicines—By John O'Reilly, M. D., F. R. C. S. I.... 112
Miner, J. F., M. D., Foreign Body removed from Left Bronchus.... 129
" Clinical Remarks upon Surgical Cases—amputation of thigh,.... 176
" Removal of Tumors, (illustration,] 178, 261
" Strabismus,.... 264
" Talipes Valgus,.... 265
" Exsection head of humurus, [illustration,].... 307
" Severe Injury—Erysipelas,.... 309
" Sero-cystic degeneration of mammary gland, [illustrated,].... 377
" Cystic Tumor of Neck, [illustrated,] 379
" Melanosis of Eye,.... 124
" Surgical Diseases of Women,.... 445
Materia, Medica and Pharmacy—By Joseph Carson, M. D., &c.... 157
Mercantile College.... 160
Manual on Extracting Teeth.... 279
Miner, J. F., M. D., Tumor of Neck—Admission of Air into Vein.... 336
McMunn's Elixir of Opium.... 338

Nitrate of Silver, use of.... 102
Nervous Force,.... 185
New York Academy of Medicine,.... 237
New York State Medical Society, transactions of for 1863,.... 237
New York State Medical Society, fifty-seventh session,.... 315
New York Medical Independent,.... 359
Nervous and vascular connection between mother and child—By John O'Reilly, M. D.... 362
New Instrument for Photographing the Eye—By A. M. Rosebrugh, M. D.... 365
Nævus, death from.... 390
Newly discovered disease depending upon Trichina Spiralis in Erie County,.... 43

Operations for hard Cataract,........... 62
Obstetrics, Hodge on..................... 477
O'Reilly—Gastrotomy,.................... 76
O'Reilly—Modus Operandi of Medicines, 112
Operation for Cataract—By J. F. Miner, M. D. ................................ 217
Operation for Varicose Veins—By J. F. Miner, M. D.......................... 219
Operations upon the Eye—By J. F. Miner, M. D......................................... 1
Ohio State Medical Society,............... 239
Opium, McMunn's Elixir of........... 338, 402
Operation for removal of head of Thigh-Bone—By J. F. Miner, M. D.......... 298
Ophthalmoscope, Dr. Rosebrugh's........ 473

Pitting in Small Pox, ................ 21
Perineum—Laceration of................. 30
Professorships—new...................... 31
Packard's Minor Surgery, ................ 37
Photography, and Murder ................ 60
Providence Insane Asylum ................ 66
Pharmacopœa, United States............. 78
Physicians, Wanted in Norfolk............ 119
Port Wine, Substitute for................. 120
Pulse Breath............................ 147
Peters, Joseph A., M. D., Spontaneous Salivation...........................95, 183
Peters, Joseph A., M. D., Cancer.......... 255
Presentation to Dr. Eastman ............ 194
Principles and Practice of Ophthalmic Medicine and Surgery—By T. Wharton Jones, F. R. S.................... 196
Parrish's Treatise on Pharmacy.... ...... 322
Proceedings Pharmaceutical Society...... 323
Personal Notice................... ...... 324
Physiology, Dalton's.................... 360
Puerperal Convulsions—By Robert Taylor, M. D.............................. 411
Physical Examination and Signs.......... 5
Pregnancy, without Emissio-Penis........ 470

Quinine, Therapeutical Action of....31, 61
Quacks in England ......... ..... ..... 120
Quack Medical Literature in Religious Family Newspapers................. 346
Quinine, Action of...................... 444

Report of Deaths................40, 402, 444
Recipe for Rheumatism.................. 96
Removal of Lower Maxilla................ 160
Report upon Epilepsy—By H. Lassing, M. D., New York ....................161, 201
Retreat for Intemperate Women........... 193
Ray, J., M. D., Mental Hygiene........... 198
Removal of Tumors—By J. F. Miner, M. D. 261
Rheumatism, Acute...................... 272
Rheumatism, Employment of Sulphur in... 391
Report, Health Physician's......40, 402, 444, 480
Rosebrugh, A. M., M. D., New Instrument. 365
Rochester, T. F., M. D., Digitalis and Veratrum .......................................... 4
Rochester, T. F., M. D., Delirium Tremens, 173, 257.

Small-Pox, to prevent pitting........... 21
Sarracenia Purpurea in small pox,........ 59
Small Pox in Washington,................ 195
Spotted Fever—By E. W. Jenks, M. D..... 81
Storer, Horatio H., M. D., artificial dilatation of os and cervix uteri,............ 114
Stretcher, field and hospital,............ 118
Surgeons for colored regiments, .......... 119
Substitute for Port Wine,................ 120
Spontaneous Salivation,................95, 132
Spontaneous Sativation again,............ 183
Students at Medical Schools,...... ....... 190
Spontaneous Salivation—Medicus,....132, 226
Surgeon General,........................ 233
Sanitary Commission U. S................ 234
Scott, Andrew J., M. D., dysentery in Ohio 241
State Board of Examiners for the Degree of Doctor in Medicine, .............. 275
Specialists,............................ 354
Specialties and Specialists — By Julius Homberger, M. D........................ 380
Specialties in Europe and America,...... 393
Surgical Diseases of Women—Inflammation Cervix Uteri—By J. F. Miner, M. D............. .................. 445

Taxis in Hernia. ............ ......... 30
Treatment of Diphtheria,................ 186
Transactions of New York State Medical Society, 1863........................ 237
Treatment of Delirium Tremens.......... 265
Treatment of Diarrhœa and Dysentery,.. 269
Treatment of Acute Rheumatism,......... 272
Tribute of Respect to Memory of Dr. Wm. H. Butler,......................... 319
Tumor in the Neck,...............226, 336, 379
Tumor, Cystic of Neck, .................. 379
Tumor, Cystic, of mammary gland,....... 377
Trichiasis in Germany,................... 383
Transactions Amer. Medical Association, 401
Taylor. Robert, M. D , Puerperal Convulsions, ................................ 411
Tracheotomy............................ 48
Tomb of Secrets......................... 470

Urine, examination of................. 20
U. S. Laboratory, Philadelphia, .......... 23
University of Buffalo, dissecting term,... 80
Uterine Pain, hypodermic treatment...... 413
Urine in Hepatic Disease,................ 443
Uterine Inflammation..................... 445

Vesico-Vaginal Fistula,...... ....... 28

Visiting List. Physicians.. .... ......... 80

Veratria in Rheumatism,..... ......... 145

Women, retreat for ................ 193

White, Prof. J. P., Diabetes,............. 373

" " Pelvic Hæmatocele......... 3

" " Seminal Emissions.......... 7

Web-fingered child, with extra toes—By O. L. Abbey, M. D..... ......... .. 383

### BOOKS AND PAMPHLETS RECEIVED.

*A Treatise on Hygiene with special reference to Military Service. By* WM. A. HAMMOND, M. D., *Surgeon-General U. S. Army; Fellow of the College of Physicians of Philadelphia; Member of the Philadelphia Pathological Society; of the Academy of Natural Science; of the American Philosophical Society; Honorary Corresponding Member of the British Medical Association; Member of the Verein Fur Gemin-schaftliche Arbeiten Zur Furderung Der Wissenshaftlichen Heilkunde; late Professor of Anatomy and Physiology in the University of Maryland; late Surgeon to and Lecturer on Clinical Surgery at the Baltimore Infirmary, etc., etc. Philadelphia:* J. B. LIPPINCOTT & Co., *Nos.* 715 *and* 717 *Market street*, 1863.

*The Pharmacopœia of the United States of America. Fourth Decennial Revision. By authority of the National Convention for revising the Pharmacopœia, held at Washington, A. D.* 1860. *Philadelphia:* J. B. LIPPINCOTT & Co., 1863.

*Braithwaite's Retrospect; a half-yearly Journal of Practical Medicine and Surgery, containing a retrospective view of every discovery and practical improvement in the Medical Sciences, digested from the leading Medical Jaurnals of Europe and America.*

This invaluable compendium, which was commenced in 1840, is issued *simultaneously* with the London edition, by virtue of an arrangement entered into with its distinguished editor, and appears regularly in *January* and *July* of each year.

*Gastrotomy—Large Abdominal-Uterine Tumor, extirpated by* JOHN O'REILLY, M. D., F. R. C. S. I. *Reported by* RICHARD J. HALTON, L. R. C. S. I. *New York: Printed by* ROBERT CRAIGHEAD, 1863.

*Annual Announcement and Circular—Bellevue Hospital Medical College, New York,* 1863–4.

For particulars notice advertisement sheet.

*Announcement of the Rensselaer Polytecnic Institute, Troy, N.Y.*, 1863–4.

The Rensselaer Polytecnic Institute aims to educate young men intending to follow the Scientific Professions, and more particularly such of them as are promotive of the leading material pursuits of the age. Its principal instruction lies, therefore, in the Departments of Chemistry, Geology and Mineralogy, Botany, and the Mathematical and Physical Sciences.

Young men, also, not contemplating active professional duty, but desirous of increasing their attainments by a partial course of scientific study, are afforded advantages which perhaps no other Institution in the country offers.

*The Atlantic Monthly, devoted to Literature, Art and Politics, August,* 1863. *Boston:* TICKNOR & FIELDS.

*Bound Volumes of the Atlantic.*—The eleventh volume of the "Atlantic," comprising January to July, 1863, is now ready, neatly bound in muslin.

